# Effect of inherent tibial asymmetry on leg length discrepancy measurements after intramedullary nailing of comminuted femoral shaft fractures

**DOI:** 10.1051/sicotj/2018053

**Published:** 2019-01-15

**Authors:** Ian Hudson, Krystalyn Mauch, Meg Schuurman, Muhammad T. Padela, Petra Gheraibeh, Rahul Vaidya

**Affiliations:** Department of Orthopaedic Surgery, Detroit Medical Center 4201 St. Antoine, Detroit MI 48201 USA

**Keywords:** Leg length discrepancy, Femur fracture, Intramedullary nail, CT scanogram

## Abstract

*Introduction*: Locked intramedullary nailing (IMN) is the standard treatment for femoral shaft fractures in adults with high rates of union and relatively low rates of complications. Leg length discrepancy (LLD) after IMN of femoral shaft fractures is common, and is reported in 20–43% of cases. A known surgical challenge when trying to obtain equal leg lengths is comminuted fracture, which results in a loss of bony landmarks that guide reduction. The purpose of this study was to assess the effect of inherent tibial asymmetry on LLD measurements after IMN.

*Methods*: Postoperative CT scanograms were performed on 79 consecutive patients after locked IMN for comminuted femoral shaft fracture. Leg lengths were determined by measurements taken from the scout view of a CT scanogram. Calculations of discrepancy were made for both femurs, tibias, and total leg length. Assessment was also made on the frequency wherein the tibial discrepancy compounded the femoral discrepancy. In situations where a limb segment was exactly symmetric to the contralateral side, the total leg was not regarded as a having compounded asymmetry.

*Results*: Notable discrepancies were found in tibial length that significantly departed from the null of symmetry (*p* < 0.0001). Forty-two patients (53.2%) were found to have a tibial asymmetry of 3 mm or more, and 20 patients (25.3%) were found to exhibit a difference of 6.3 mm or more. Median femoral discrepancy was 5.3 mm and median tibial discrepancy was 3.0 mm. Seven patients were found to be asymmetric in total leg length as a consequence of underlying tibial asymmetry. Conversely, 11 patients benefited from their tibial asymmetry, which compensated for femoral asymmetry after IMN.

*Conclusion*: Tibial symmetry cannot be assumed. If not accounted for, inherent tibial asymmetry may influence LLD after IMN of femur fractures.

## Introduction

Leg length discrepancy (LLD) after locked intramedullary nailing (IMN) of femoral shaft fractures is common, reported in 20–43% of cases, and is particularly a problem in comminuted fractures [1–4]. LLD may cause an asymmetric gait, lead to degenerative arthritis of the knee, hip, and/or lumbar spine, and may require corrective surgery or shoe modifications. Iatrogenic LLD may lead to litigation and patient dissatisfaction, and is the second most-cited source of medical malpractice litigation among members of the American Association of Hip and Knee Surgeons following total hip arthroplasty [2,5–8]. An LLD of up to 2 cm has been reported to be acceptable in congenital LLD, but 1.5 cm is often troublesome for patients with iatrogenic LLD after hip replacement [9,10].

Multiple techniques are described to help preclude LLD intraoperatively during femur fracture fixation such as using navigation systems, sterile bovie cords, radiopaque rulers, and traction but LLD still occurs [2,4,11–14]. Fluoroscopic techniques may not be accurate to 1.5 cm due to leg swelling, and the inability to do long leg views [15]. Postoperative assessment of leg lengths can be done by orthoroentgenography, MRIs, and CT scanograms. CT scanograms have been shown to be accurate with good intra observer reliability to measure LLD and femoral version that can be accomplished with low radiation dose protocols [2,4,13,14,16,17]. Many studies use femoral length on CT scanograms to ensure the correct leg length was achieved after fixation, while ignoring any tibial contribution [4,5,18–20].

The purpose of this study is (1) to assess if inherent tibial asymmetry corrects, aggravates, or had no effect on the calculated measurements of LLD after IMN for comminuted femoral shaft fracture, and (2) if tibial measurements are necessary to minimize LLD.

## Materials and methods

This Institutional Review Board approved prospective study includes 79 consecutive patients who underwent a postoperative CT scout scanogram after locked IMN for a comminuted femoral shaft fracture at our Level 1 hospital between 2012 and 2016. The patients were enrolled in a quality control improvement project that sought to prevent patients from leaving our hospital with an iatrogenic LLD [15]. All patients had comminuted femur fractures, AO/OTA classification Type B (20), or Type C (59) patterns. Their age ranged from 16 to 94 with mean of 33.4 years. There were 67 men and 12 women. The common reasons of injury were motor vehicle accident (30), gunshot injuries (45), and falls (3). Patients were taken to surgery within 24–48 h from admission. Forty-four patients were treated with antegrade nails and 35 with retrograde nails. All patients received a statically locked nail. Surgeons used either a bovie cord or radiolucent ruler during surgery to establish length compared to the normal leg.

Postoperative CT scanograms were taken during each patient's hospital stay, typically the first day after surgery. The CT scanograms were performed with the patient lying supine on the CT table with both hips and knee in extension. AP and lateral scout images were taken to measure the lengths of the operative and nonoperative femurs, tibias, and total leg lengths. Lateral scans are done to make sure that the two limbs are in perfect alignment in the sagittal plane, i.e., one leg is not flexed at the hip or knee when we do the CT scanogram. The CT technician in the case of a patient being unable to make their leg perfectly straight uses blankets in the normal limb to establish the same sagittal alignment as the operated limb. Femur lengths were measured from the superior aspect of the femoral head to the most distal portion of the medial femoral condyle. The length of each tibia was measured from the tibial eminence to the middle of the tibial plafond. Total leg length was measured from the top of the femoral head to the middle of the tibial plafond. The CT scanogram protocol at our institution has a relatively low cost ($380), low radiation, and good interobserver reliability (Pearson's correlation coefficient *r*-value 0.9973118) [2]. A LLD >15 mm was defined as unacceptable in this study.

To quantify the level of risk accepted by an orthopaedic surgeon in failing to account for an intrinsic tibial length discrepancy when attempting to achieve overall leg symmetry, an assessment was made on the frequency wherein the tibial discrepancy compounds the femoral discrepancy. In situations where a limb segment was exactly symmetric, the total leg was not regarded as having compounded asymmetry. A nonparametric assessment of the absolute value of tibial difference was performed to determine whether tibial symmetry might be assumed (Wilcoxon sign test, null hypothesis of mean tibial difference equals zero). Statistical analysis was performed using SAS 9.3 (SAS Institute, Cary, NC). Significance was set at *p* < 0.05.

## Results

Fourteen patients (18%) were found to have a LLD > 15 mm, which we used as a cutoff for excessive and six of these patients (8%) had a LLD > 20 mm (Table 1).

**Table 1:** Descriptive statistics of demographics, surgical method, and limb characteristics.

		95% CI
Gender (male)	83.8%	
Age (mean)	33.46	(30.0, 35.6)
Fixation approach	50.6% antegrade	
Femoral mean (mm)	477.90	(473.3, 484.2)
Femoral discrepancy (median, in mm)	5.3	
Tibial mean (mm)	382.38	(377.8, 386.9)
Tibial discrepancy (median, in mm)	3.0	
Proportion with compounding	34/79 (43.0%)	(34.1, 54.0)

### Femur measurements

The mean femoral difference was 7.4 mm, while the median femoral discrepancy was 5.3 mm.

### Tibia measurements

Notable discrepancies were found in tibial lengths, significantly departing from the null of symmetry (*p* < 0.0001). The mean tibial difference was found to be 4.75 mm, but the median of 3.0 mm was viewed as more representative to control for a small group of outliers. Forty-two patients (53.2%) were found to have the median tibial asymmetry of 3 mm or more, and 20 patients (25.3%) were found to exhibit a difference of 6.3 or more (Table 2). Only 17 (22%) patients had no tibial asymmetry measured.

**Table 2:** Tibial discrepancy percentiles and projected population prevalence.

Percentile	Discrepancy (mm)	Number of patients	Percentage	95% CI
50th	3.0	42/79	53.2%	(42.2%, 62.2%)
75th	6.3	20/79	25.3%	(15.7%, 34.9%)
90th	12.9	7/79	8.9%	(2.6%, 15.1%)

### Total leg length measurements

The mean total leg length difference was (10 mm), while the median total length discrepancy was (8 mm). A LLD > 15 mm occurred in 14 patients (17.7%) (Table 2).

Thirty-four (43%) of the patients with intrinsic tibial discrepancy compounded the total length measurements. There was no impact from the intrinsic tibial asymmetry of seven patients on the total limb length being greater than 15mm, while the other seven patients were found to be unacceptably asymmetric in total leg length as a consequence of their underlying tibial asymmetry. Had these seven patients demonstrated tibial symmetry, their total leg lengths would have been under the surgical consideration threshold. Conversely, 11 patients benefited from their tibial asymmetry, which compensated for femoral asymmetry after IMN.

**Figure 1:**
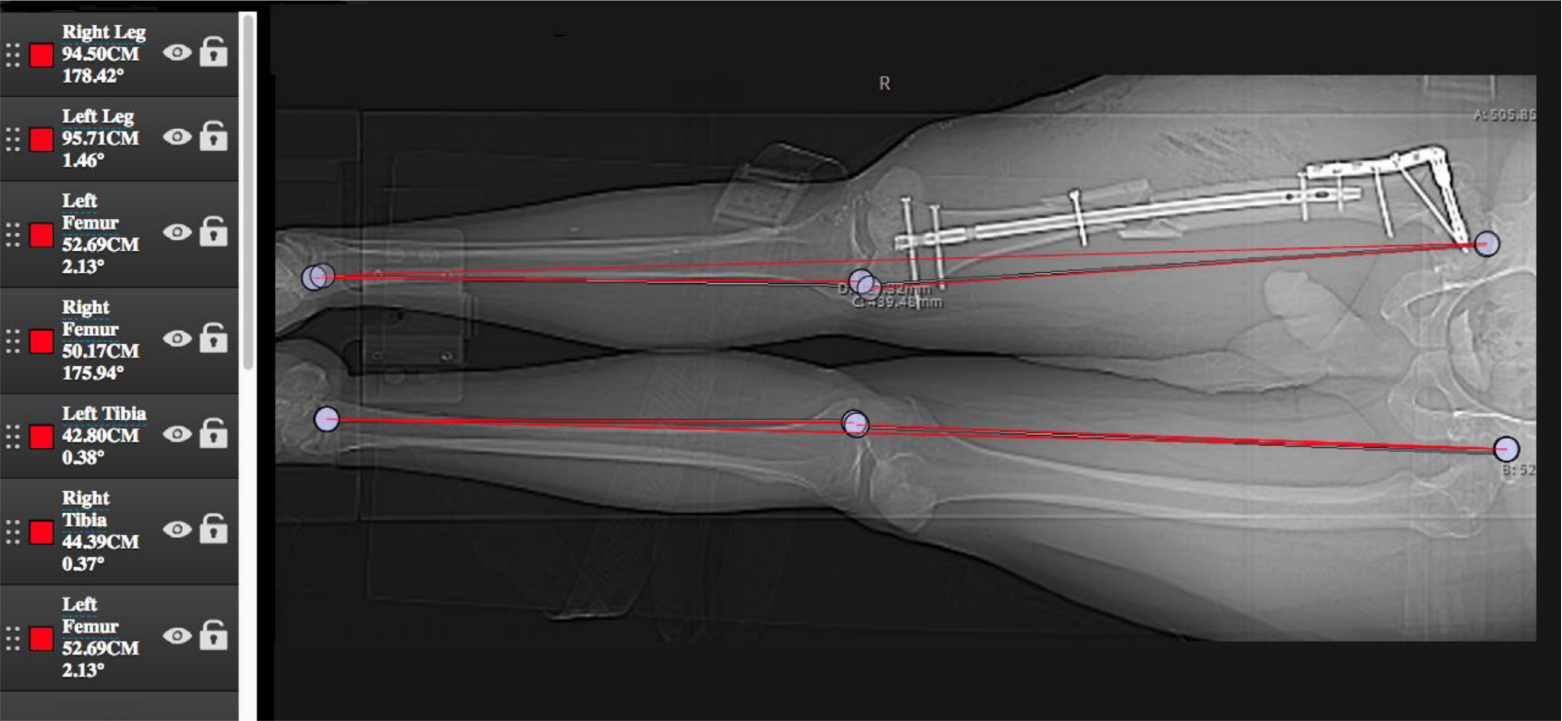
This 40-year patient with a right previous proximal femoral fixation and new right comminuted femur fracture appears to be nailed with a −2.42 cm (shortening) of this right femur compared to the left femur. However, due to a Tibia LLD of +1.59 cm (inherently longer) on the right side, he has a balancing of the overall LLD of <0.5 cm, which is and was tolerable for him.

**Figure 2:**
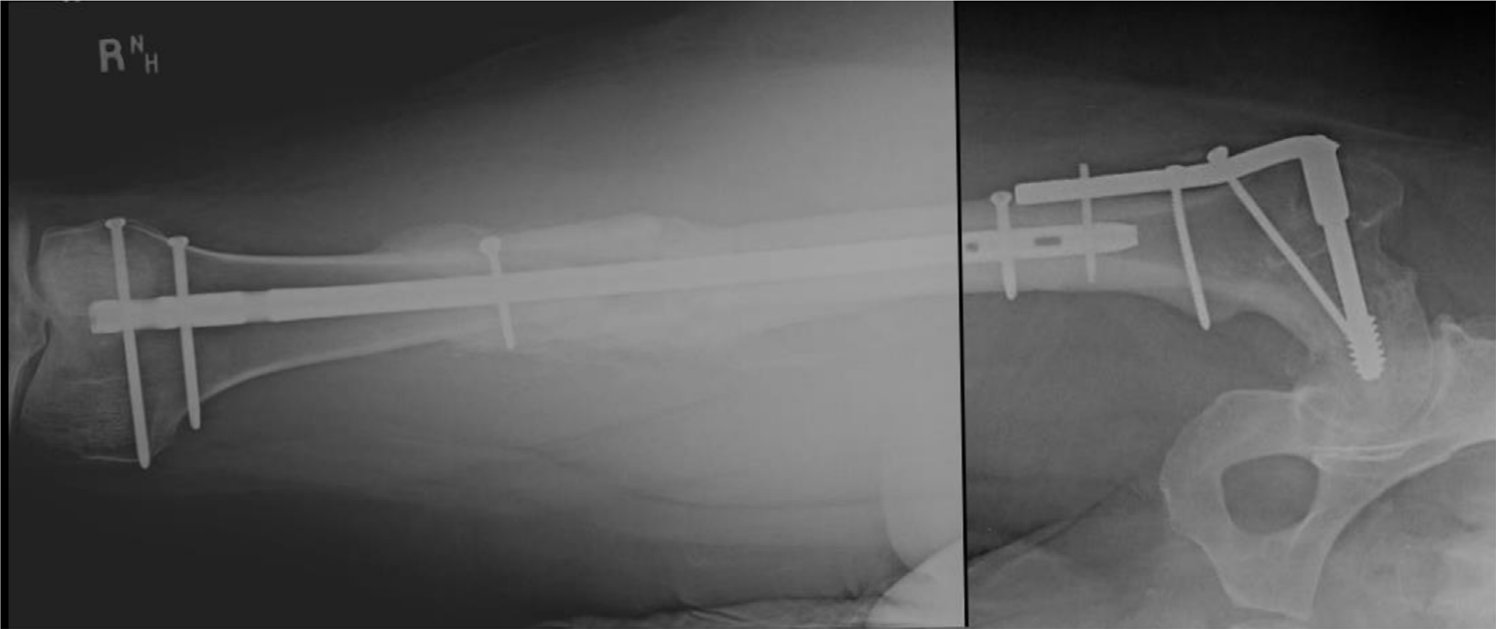
Healed fracture with no symptoms of leg length inequality.

Thus, 18 patients had a LLD that was impacted by tibial asymmetry that either brought them into the unacceptable (>15 mm) or corrected their femoral LLD to an acceptable (<15 mm) asymmetry (Figures 1 and 2).

## Discussion

IMN is the gold standard for treating femoral shaft fractures with high rates of union and low rates of morbidity; however, LLD remains common [3,5]. If bony landmarks are lost to comminution, it can be challenging to restore length. LLD can occur even with multiple methods used intraoperatively to assist leg length restoration such as applying traction, external distraction devices, navigation systems, bovie cord, or radiopaque rulers [2–4]. A postoperative CT scanogram is a preferred method to obtain accurate measurements of limb length [2–5,14] and can be performed with low-dosage radiation protocols [17]. At our institution, a CT scanogram costs $380, while a femur X-ray costs $260. We use a strict positioning protocol to obtain a CT scanogram and assess lateral scout images to ensure there is no flexion of the hip or knee to confound our measurements, allowing us to obtain relatively low cost, low radiation, and accurate measurements of our patients' bilateral femurs, tibias, and total leg lengths to measure LLD after fixation of a comminuted femur fracture. In this study, we used these measurements to see if inherent tibial asymmetry played a role in our calculated LLD measurements after IMN comminuted femoral shaft fractures, and whether it influenced those measurements positively or negatively.

Small limb length discrepancies of the lower limbs are a common clinical finding that can be physiological. LLD is found to be a normal variant in up to 70% of the general population, and the US army observed 51% of recruits had a discrepancy of less than 5 mm [2,21]. Strecker et al. [22] found inherent asymmetry between limbs had a median difference of 3 mm, which increased to 9 mm in the 95th percentile. Literature suggests that the amount of LLD considered clinically significant is controversial and small differences may be asymptomatic. We were unable to ascertain if any of the 79 patients in our study had inherent total leg length asymmetry prior to injury.

Acute discrepancy in limb length may be less well tolerated than a congenital one and can cause significant morbidity. An acute LLD with as little as 5 mm is reported to be associated with back and hip pain, while a simulated discrepancy of 10 mm causes significant increase in postural sway [1,6,18,21,23]. Betsch et al. [6] reports that LLD >20 mm leads to significant changes in spinal posture. LLD also alters gait mechanics since the longer limb has increased pressures in the push-off phase, and degenerative changes in articular cartilage may occur under increased load levels [11,12]. Stride mechanics and pain of the knee, hip, or back may all be affected by LLD over time. With long-standing LLD, it is important to consider that differences in leg length may not only be due to bony length. Contractures of lower extremity joints, pelvic obliquity, and/or muscle imbalances can alter perceived discrepancies in limb length. It is important to account for these factors in clinical examinations and to check the lateral scout CT scanogram to rule out contractures that may alter measurements of length in an AP view. Further kinesiologic discussion on the potential for excess wear of one or both lower extremity joints due to unaccounted limb asymmetry “under threshold” and an artefact of compensating for femoral and tibial discrepancies is beyond the scope of this article. However, it does merit consideration in future studies.

Previous studies use 15 mm as a theoretical cutoff to revise iatrogenic LLD [1,2,4,18,19,24]. It is reported that normal inherent differences in femoral length can be up to 12 mm [25]. Winquist et al. [1] report patients who had less than 20 mm of shortening also had minimal back and hip pain. In patients older than 65, <25 mm of LLD was acceptable and in younger patients <15 mm of LLD was acceptable.

**Figure 3:**
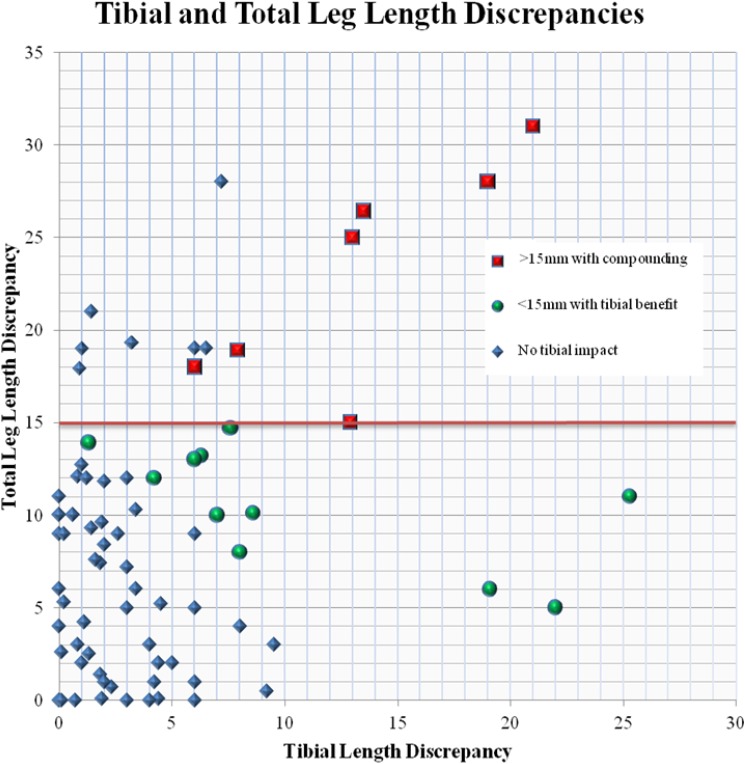
Leg length discrepancies compared to tibial length discrepancies. Leg disparities above the 15 mm threshold that were compounded by intrinsic asymmetry of the tibias are shown in red, while those with leg length discrepancy less than 15 mm only due to tibial asymmetry are in green.

In this investigation, we noted 14 patients (17.7%) demonstrated a LLD of >15 mm after the index procedure and 6 of those patients were >20 mm (7.6%). It is our belief that significant LLD should be corrected in the same admission as the initial procedure since simply returning to the operating room to relock the nail at the correct length may be done more readily if the fracture has not healed. This may avoid having to wear a shoe lift or, in some cases, avoid a more extensive surgical procedure like lengthening or shortening of the contralateral leg. In a previous study at our institution, it was noted that tibial lengths were unequal in a majority (89%, 25 of 28) of patients and contributed to total LLD [2]. Subsequently, we found that 34 (43%) patients had compounding measurements of total limb length. Eighteen patients' (22.7%) total LLD measurements affected our revision treatment guidelines in this cohort because of the compounding effect of the tibial measurements. Eleven patients benefited from their inherent tibial asymmetry, while the other seven patients were negatively influenced (Figure 3). On the other hand, seven patients over the 15 mm cutoff had no impact from their tibial asymmetry. Fifty percent of those potentially needing revision of their femoral IMN (LLD > 15 mm) had nontrivial contributions due to inherent tibial asymmetry. The remaining 50% had significant discrepancy due to their inherent tibial asymmetry. Statistical significance is not equivalent to clinical significance, and we acknowledge that a 3 mm median tibial discrepancy seems small. However, this 3 mm median discrepancy matched the 3 mm inherent asymmetry difference in limbs found in Strecker's et al. [22] length and torsion study of the lower limb. It is our opinion that frequent and potentially large differences between tibias must be considered before correcting a LLD after a femoral fracture because the aim of the fracture fixation should be to obtain equal limb lengths. In Figure 3, three outliers of tibial length are identified with discrepancy between the paired tibias over 18 mm, which can be accounted for if the total leg length is used to determine length instead of just femoral length.

One of the major limitations of this study is our inability to ascertain if the patients in this study had inherent total leg length asymmetry prior to injury and whether it is wise to have final LLD of zero or back to what it was originally.

In conclusion, total leg length should be the measurement that we use to determine LLD after femoral nailing and not just femoral length. We have shown that there exists a propensity for asymmetric tibial length to affect total leg length adversely or to benefit a LLD in the femur after IM nailing of a femur fracture.

## Conflict of interest

No funding was received in the production of this paper or as a grant for this publication.

Ian Hudson certifies that he has no financial conflict of interest in connection with this article.

Krystalyn Mauch certifies that she has no financial conflict of interest in connection with this article.

Meg Schuurman certifies that she has no financial conflict of interest in connection with this article.

Muhammad T. Padela certifies that he has no financial conflict of interest in connection with this article.

Petra Gheraibeh certifies that she has no financial conflict of interest in connection with this article.

Rahul Vaidya has the following conflict of interest disclosures:

European Spine Journal: Editorial or governing board

JOT editorial Board

Smith & Nephew: IP royalties, paid speaker

Smith and Nephew Institutional Research Grant Funding

Depuy Synthes: IP royalties

AO Foundation Paid Speaker
